# Effect of Fabrication Route on the Mechanical Properties of Polylactic Acid (PLA) Composites with Diatom Earth (DE)

**DOI:** 10.3390/polym17162208

**Published:** 2025-08-13

**Authors:** Adrian Dubicki, Magdalena Pantoł, Krzysztof Jan Kurzydłowski

**Affiliations:** 1 Institute of Mechanical Engineering, Bialystok University of Technology, Wiejska 45C Street, 15-351 Bialystok, Poland or magdalena.pantol@grupaazoty.com (M.P.); k.kurzydlowski@pb.edu.pl (K.J.K.); 2 Grupa Azoty S.A., Biopolymer Centre, Kwiatkowskiego 8 Street, 33-101 Tarnów, Poland

**Keywords:** polylactide (PLA), diatomaceous earth (DE), polymer composites reinforced with silica, biodegradable composites, injection molding (IM), 3D printing, biomaterials, mechanical properties

## Abstract

Polylactide (PLA) that is reinforced with diatomaceous earth (DE) is a promising and eco-friendly material with high engineering potential. This article provides a comprehensive overview of various PLA types and processing methods for PLA + DE composites. This study aimed to determine the mechanical strength limits of PLA + DE composites using two PLA grades—amorphous PLE 005-A and semi-crystalline Ingeo 4043D—that are each filled with Perma-Guard DE 5, 10, and 15% by weight, and two manufacturing methods, injection molding (IM) and additive manufacturing (3DP), using fused filament fabrication (FFF). The mechanical properties were assessed through static tensile tests in accordance with ISO 527-1 and compared with values reported in the literature. The results indicate a linear increase in stiffness (Young’s modulus) with increasing DE content. This is accompanied by a reduction in maximum tensile strength (σ_max_) and elongation at break (ε_b_). The highest Young’s modulus, around 4.65 GPa, was observed for injection-molded, semi-crystalline PLA with a 15% by weight DE. The greatest tensile strength, approx. 72 MPa, was achieved for printed, semi-crystalline PLA without filler. Furthermore, 3D printing achieved a tensile strength and stiffness comparable to injection molding, though the latter ensured significantly better ductility. These findings provide a basis for adjusting the PLA + DE composite properties to specific applications by selecting the matrix type, DE content, and manufacturing method.

## 1. Introduction

Polylactic acid is a polymer (an aliphatic polyester), commonly known as polylactide (PLA) [1]. The monomer is obtained by fermenting sugars derived from natural sources such as corn, sugar beets, or sugarcane, making it an eco-friendly alternative to petrochemical-based plastics. Industrial practice for PLA production favors the ring-opening polymerization (ROP) of lactide because it allows for precise control over the polymer’s molecular weight and stereostructure. The direct polycondensation of lactic acid is not preferred, mainly due to the challenges in managing its molecular weight [1,2,3].

The properties of polylactide depend particularly on its molecular weight (Mw) but also on its stereochemical composition, which directly influences crystallinity. In PLAs with a stereochemical structure that is based solely on L-lactide (PLLA) or D-lactide (PDLA), there is a tendency to crystallize, although this process is slow. Increasing the proportion of either enantiomer accelerates crystallization [2,4]. Stereocomplexes of PLA can then form, with a degree of crystallinity (Xc) exceeding 50% when the enantiomers are present in equal amounts [4,5,6]. The amorphous variant of PLA is regarded as PDLLA, which is produced by polymerizing racemic D,L-lactide [2]. The variety of PLA stereochemical configurations thus translates directly into a wide range of physical properties for this material.

Polylactide is a thermoplastic whose glass transition temperature (Tg) ranges from approximately 50 to 70 °C, while its melting temperature (Tm) is about 150–180 °C [2,5]. When a PLLA/PDLA stereocomplex (scPLA) forms, the melting temperature increases by roughly 50 °C [6]. The mechanical properties of polylactide are also highly variable and strongly dependent on molecular weight [2]. In its basic semi-crystalline form, this plastic is generally regarded as rigid, moderately strong, and brittle. Its tensile strength ranges from about 50 to 70 MPa, dropping to as low as 30 MPa when the amorphous phase is fully dominant [2]. Young’s modulus is reported as 0.4–3.5 GPa for variants that are considered amorphous, and 2.7–4.2 GPa for semi-crystalline variants [1,2].

**Figure 1:**
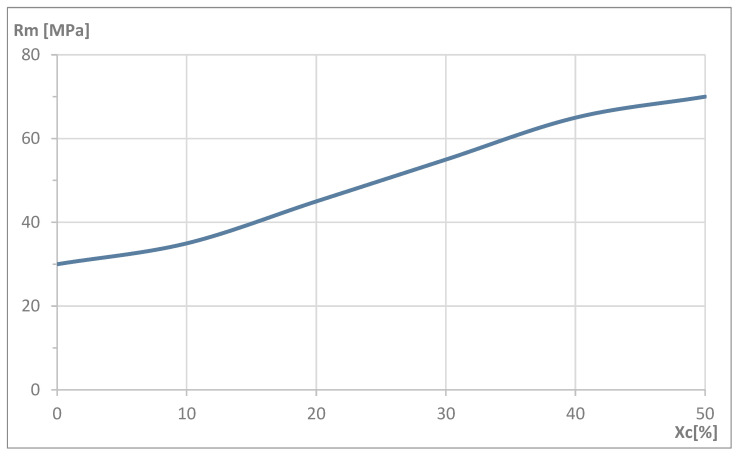
Approximate relationship between the tensile strength level (Rm) and the degree of crystallinity (Xc) of polylactide (PLA) [1,2,4,7].

In general, assuming that there is no change in the molecular weight, an increase in crystallinity leads to improved mechanical properties such as tensile strength, elastic modulus, and stiffness. However, a higher degree of crystallinity negatively affects elongation at break, reducing the material’s plasticity and making the material more brittle [2,4]. The maximum crystallinity level (Xc) of PLA differs for the PLLA and PDLA variants and reaches up to 46% and 49%, respectively [7]. The crystallinity of the stereocomplex can reach a maximum of 50–55% [5]. Considering the tensile strength level of amorphous PLA and its increase as it transforms into semi-crystalline and stereocomplex PLA, an approximate graph (Figure 1) has been developed and presented, showing the relationship between tensile strength (Rm) and the degree of crystallinity (Xc). It can be concluded from the data in Figure 1 that the upper bound for the tensile strength of high crystallinity PLA is 70 MPa. In fact, it agrees well with the results of our study, which is presented in the following text.

Considering the information presented above, two types of polylactide were used for our research purposes: semi-crystalline PLA Ingeo 4043D (NatureWorks LLC, Plymouth, MA, USA) and amorphous PLE 005-A (NaturePlast SAS, Mondeville, France). The PLA Ingeo 4043D granulate represents a semi-crystalline PLA variant with slow crystallization kinetics. It is commonly used in the extrusion of filaments for 3D printing, providing good adhesion, stiffness, limited shrinkage, and a balance between rigidity and strength [8]. The amorphous PLA used in this study is PLE 005-A granulate. The manufacturer recommends this material for injection molding, thermoforming, or extrusion. It is characterized by higher impact resistance and better ductility, but with lower stiffness [9].

When comparing the materials, Ingeo 4043D has a slightly higher glass transition temperature (approx. 55–60 °C) than PLE 005-A (51 °C). The semi-crystalline PLA variant shows a melting temperature in the range of 145–160 °C. The tensile strength of Ingeo 4043D is about 53 MPa, while for PLE 005-A it is lower, at approximately 45 MPa. A similar relationship occurs in Young’s modulus, which for the above-mentioned materials is 3600 MPa and 3500 MPa, respectively. In general, both variants fall within similar and comparable ranges, with a slight advantage in mechanical properties and thermal resistance for PLA Ingeo 4043D. The melt flow rate (MFR) of both materials is determined to be around 6 g/10 min. The MFR is strongly correlated with the polymer’s molecular weight, which allows us to state that both of the PLA variants used have very similar molecular weights. In the conducted studies, the main factor determining the mechanical properties of the two types of PLA used is the level of polylactide crystallinity [8,9].

Diatomaceous earth (DE), also known as diatomite, is a silica-based soft sedimentary rock of organic origin. It is composed mainly of the microscopic shells of dead unicellular algae that belong to the diatom class. Its chemical composition is primarily based on silica (SiO_2_), with smaller amounts of aluminum oxide (Al_2_O_3_), calcium oxide (CaO), magnesium oxide (MgO), iron oxide (Fe_2_O_3_), and remnants of organic matter [10,11]. The growth of diatoms—and therefore the final composition of diatomaceous earth—depends on environmental factors such as the availability of dissolved minerals, light, and salinity [11].

Diatomite is characterized by a particular combination of chemical and physical properties. This translates into a wide spectrum of applications, from agriculture, through industry and medicine, to construction [10,11]. Thanks to its high porosity, well-developed specific surface area, chemical inertness, and high permeability, diatomaceous earth serves as an effective filtration medium, which is used to purify wine, beer, oil, or vinegar [12]. Diatomaceous earth also finds broad applications in pharmacy and medicine. It is known as a supplement that supports body detoxification, and it improves the health condition of skin, hair, nails, and the skeletal system [13]. Diatom frustules function in medicine as carriers for drugs released in a controlled manner or as components of dressings that aid wound healing by absorbing exudates and delivering bioactive substances. In implantology, when combined with hydroxyapatite (HAp), it is employed to produce biodegradable polymer implants that positively influence the osteointegration processes and bone tissue regeneration [14].

Low thermal conductivity makes diatomaceous earth useful in concrete mixtures and thermal-insulation building materials as well [10,15]. Research into the use of diatoms in electrotechnics—for the production of high-performance capacitors and batteries—may prove to be exceptionally interesting and groundbreaking [16].

Owing to its natural origin, biodegradability, and unique properties, diatomaceous earth is an appealing functional material and a desirable component for engineering applications. In this study, amorphous diatomaceous earth Diatomit (Perma-Guard Inc., North Salt Lake, UT, USA) was used. The material originates from fossil deposits of *Melosira preicellanica* diatom frustules located in New Mexico (USA), which is noted for its exceptional purity and quality. It contains less than 0.5% crystalline phase, and its main constituent (approximately 92%) is silica (SiO_2_) [17].

Blending polylactide (PLA) with additives alters the material’s properties—improving thermal stability and resistance, accelerating crystallization, increasing mechanical strength, and enhancing biocompatibility [2,3,4,7]. The effects of a given property depend on the type of additive that is introduced into the PLA matrix and its structural form. A comprehensive literature review by Ranakoti et al. (2022) concluded that appropriately selected and, in many cases, modified additives produce significant changes in the mechanical and thermal properties of PLA [4]. Greater thermal stability of the composite is achieved with the addition of silica or titanium dioxide. These additives improve resistance to degradation by raising both the glass transition temperature and the melting point. The enhanced PLA crystallization rate, which directly translates into increased material strength, was noted with the addition of carbon nanotubes, rice husk flour, and silica that was modified with oligomers of lactic acid. Additions of silica or calcium carbonate can even double the composite elastic modulus [4].

## 2. Materials and Methods

To investigate the influence of diatomaceous earth (DE) on the properties of a PLA-based composite—particularly the tensile strength and stiffness—several variants of PLA + DE composites were prepared. Two grades of PLA granulate were used as the matrix: amorphous PLE-005-A and semi-crystalline PLA Ingeo 4043D, with each filled with diatomaceous earth diatomit at weight fractions of 5%, 10% and 15%. The composition was supplemented (at the expense of PLA mass) with 0.3% calcium stearate as a processing aid in polymer manufacturing.

A multi-stage twin-screw extrusion system was used to produce the PLA + DE composite material. Processing was carried out at temperatures between 176 °C and 188 °C and at pressures from 3.8 to 4.4 MPa. The extruded fiber was cooled in water and air, and then pelletized and dried for 8 h at 60 °C before further processing.

Specimens were produced by two manufacturing methods, injection molding (IM) and additive manufacturing, commonly known as three-dimensional printing (3DP). Using these methods, samples were made from pure PLA (PLA 4043D or PLE 005-A) and from composites containing 5%, 10%, and 15% diatomaceous earth by weight. Accounting for both processing techniques and material compositions, 16 specimen variants for further testing resulted.

Injection molding was performed on an Engel Victory 50/200 (Engel GmbH, Schwertberg, Austria) laboratory injection molding machine. The processing temperatures for the PLA 4043D-based variants ranged from 170 °C to 200 °C, while for the PLE 005-A-based variants, they ranged from 170 °C to 185 °C. The molding pressure for the composites reached a maximum of about 12 MPa for the amorphous (PLE) matrices, and roughly half that for the semi-crystalline (PLA) matrices. Pure PLA samples, without diatomaceous earth additives, were molded at pressures of 6–7 MPa. The mold temperature was held at 40 °C throughout processing. The total cycle time for molding each test specimen was 50–55 s.

Additive manufacturing was carried out on a Creator PRO 2 (Zhejiang Flashforge 3D Technology Corporation, Jinhua, China) using fused filament fabrication (FFF). Prints were produced from extruded polymer and composite filaments of 1.75 mm diameter. A 0.6 mm nozzle—which is recommended for composites—was used, with a 100% infill (perimeter fill) and a layer height of 0.2 mm. To ensure inter-road adhesion, the extrusion multiplier was experimentally increased to 115%. The nozzle temperature was set to 195 °C, and the build-plate temperature to 45 °C. The fabrication of a single specimen took approximately 55 min. To minimize the influence of anisotropy and achieve a maximum print strength, the specimens were oriented horizontally on the build plate, with the extrusion paths running along the long axis of the specimen [18,19].

For testing the mechanical properties of the material, standard tensile strength samples (dog-bone samples) were prepared and were dimensionally compliant with the ISO 527 standard (Plastics—Determination of tensile properties, CEN: Brussels, Belgium, 2012). The overall specimen length was 170 mm. The gauge section measured 75 mm in length, 10 mm in width, and 4 mm in thickness. The fillet radius between the grip and gauge sections was approximately 25 mm. This geometry ensured a uniform strain distribution across the gauge area and yielded reproducible and comparable strength test results.

To determine the tensile properties of the specimens, a dynamic biaxial testing machine, the MTS 858 Mini Bionix (MTS Systems Corporation, Eden Prairie, MN, USA), equipped with an extensometer, was used. Static uniaxial tensile tests were carried out at room temperature (approximately 22 °C). The initial crosshead speed was set to 1 mm/min, corresponding to a strain rate of 1.67 × 10^−4^ s^−1^. Each material variant was represented by at least six specimens.

## 3. Results

**Figure 2:**
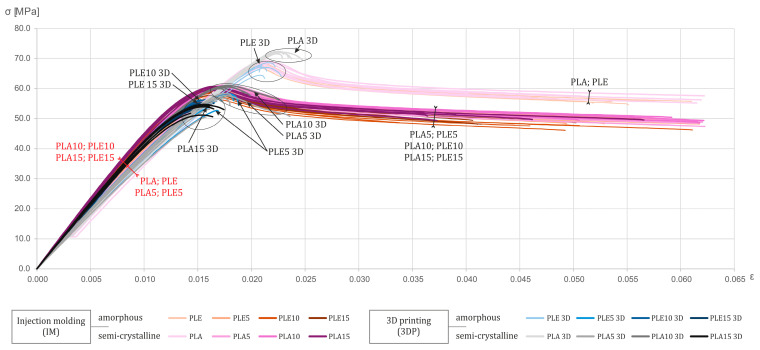
Stress–strain curves for all tested material types and compositions. Legend: PLA = semi-crystalline polylactide; PLE = amorphous polylactide; numbers = weight% DE.

The outcome of the conducted procedures and tests on the obtained materials with respect to the tensile strength was a set of results, including the stress–strain curve. These results are presented collectively in Figure 2.

**Figure 3:**
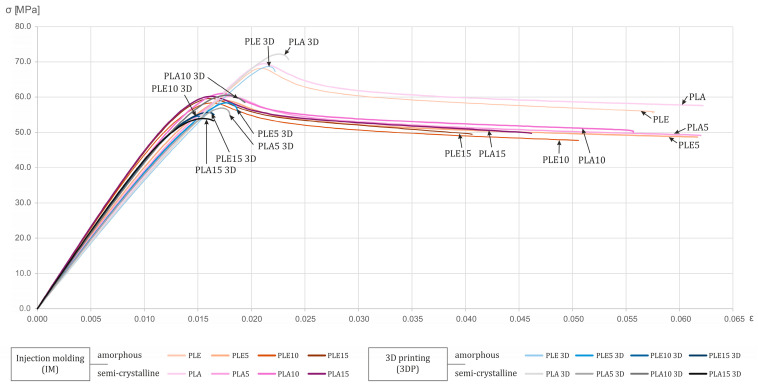
Representative stress–strain curves for all tested material types, compositions, and processing methods. Legend: PLA = semi-crystalline polylactide; PLE = amorphous polylactide; numbers = weight% DE.

For the sake of clarity in the presentation of the test results, Figure 3 also includes a separate graph of the representative stress–strain curves.

The tests enabled a comparison of various material combinations, differing in the degree of polymer crystallinity, the amount of filler introduced, and the method of specimen fabrication.

The results of the static tensile tests show that the injection-molded, unmodified, semi-crystalline polylactide PLA (Ingeo 4043D) reached a maximum tensile stress (σ_max_) of approximately 70 MPa, which is in agreement with the data presented in Figure 1. The PLE variant (PLE 005-A), considered amorphous, lost about 7% compared to the PLA, exhibiting a maximum stress of roughly 65 MPa. The difference arises from the ordered crystalline regions in the semi-crystalline material, which bridge the polymer chains and, thus, increase resistance to applied forces, also shifting the yield point [2,20].

In the composites, the introduction of diatomaceous earth filler into the polylactide leads to a sudden drop in maximum tensile stress. As the filler content increases, its strength continues to decline, but more gradually. This abrupt change is due to the stress concentration at the matrix–filler interface. At higher DE loadings, particle agglomeration of the diatom frustules further degrades strength, as confirmed in the literature [4].

Specimens that were produced by the FFF additive manufacturing maintain maximum tensile stress values like their injection-molded counterparts. However, the addition of silica-based filler further reduces strength. In FFF printing, incomplete bonding between adjacent roads and layers increases porosity, which exacerbates crack initiation and propagation.

The experimental data indicate a steady increase in the elastic modulus (E) of PLA-based composites as the DE content rises. The rate of increase, or even a slight drop, was, however, observed for the composite with the lowest DE content (5% by weight). A similar trend was reported by Zgłobicka et al. (2022) for another PLA grade (Ingeo 3001D) [21]. This behavior accords with composite theory: a stiffening filler restricts matrix deformation while maintaining structural continuity. Diatomaceous earth, as a mineral filler with high stiffness and a microporous structure, fits this model, limiting polymer-chain mobility and, thus, raising composite stiffness [21,22].

When analyzing the change in Young’s modulus in terms of the polymer or composite processing technique, a greater increase was observed in the specimens produced by injection molding. The main reason is the more uniform stress transfer, made possible by good interlayer cohesion, which translates into lower porosity. In the case of 3D printing, the indicated parameters are not at such a satisfactory level [4].

The presented graphs show that the semi-crystalline PLA variant exhibits slightly higher elasticity values. Amorphous polylactide, which is due to the absence of bridging and stiffening crystalline regions in the material, achieves lower elasticity. The same relationships can be observed in the composite variants as well. Additives in the form of diatomaceous earth positively influence the cold crystallization processes, which result in further stiffening of the material [22].

The manufacturing process used and the filler content both have a significant impact on the material’s elongation at break (ε_b_). A marked reduction in ductility was observed when 3D printing—in this case, FFF technology—was employed. Regardless of the base material or the amount of diatomaceous earth added, the printed specimens failed immediately after reaching their maximum tensile stresses. Injection-molded polylactide achieved an elongation at break of approximately 6%. In the literature, unmodified PLA’s elongation at break is reported in the range of 4–7% [2,23]. Gaps between extruded roads and layers in the 3D-printed material, as well as microcracks, contribute to earlier and more brittle failure. Injection molding, by maintaining a uniform temperature throughout the material, promotes polymer-chain entanglement and, thus, better ductility. Our tests show nearly a 40% reduction in elongation at break (from 4–6% for IM down to 1.5–2.5% for 3DP), which aligns with the literature reports of ductility losses exceeding 30% [23].

To a lesser degree, the addition of diatomaceous earth also reduces elongation. For injection-molded samples, a 5% by weight diatomite loading does not negatively affect ductility: both the pure PLA and the PLA + DE 5%/PLE + DE 5% composites retain elongation at break at around 6%. Similar observations appear in other studies [21]. However, increasing the biogenic silica content leads to a gradual drop in elongation at break to about 4%. This effect arises because the rigid diatom frustules stiffen the composite matrix yet also act as stress concentrators, initiating brittle crack propagation [4,21].

In additive manufacturing, diatomite additions likewise decrease ductility (by about 0.50–0.75%), but the dominant factor remains the chosen processing technique. In most configurations, the amorphous polylactide exhibits lower ductility than its semi-crystalline counterpart.

The conducted experiments and literature analysis provide a multifaceted view of the basic mechanical properties of polylactide and its diatomaceous earth composites. The static tensile test results indicate three main relationships:

❖ the tensile strength (σ_max_) reaches its highest value for the pure, semi-crystalline variant of polylactide (PLA), slightly exceeding the amorphous variant (PLE), and decreases in all matrix variants upon the addition of the biogenic silica filler (DE);
❖ the elastic modulus (E) increases with the growing content of diatomaceous earth (DE) in the material, with more noticeable changes observed in the specimens produced by injection molding (IM) than by 3D printing (3DP);
❖ the elongation at break (ε_b_) reaches its highest level in the injection-molded, semi-crystalline polylactide (PLA) variant without additives, while due to the specific conditions of additive manufacturing (3DP) and the brittleness of the filler (DE), the worst performance is exhibited by the FFF-manufactured PLE + DE composites with a high diatomaceous earth (DE) content.

These relationships can guide the design of engineering materials based on a polylactide matrix. It should be noted that PLA–DE composites require a comprehensive approach and balancing of benefits. Increased stiffness improves structural load-bearing capacity, but the concurrent reduction in strength and ductility limits their use in more dynamic applications. The manufacturing method also plays a key role in the final properties: injection molding allows for a fuller exploitation of filler properties, whereas additive manufacturing offers greater freedom to produce complex geometries and lower costs for small-series production despite a lower reliability and durability of the printed parts.

Optimizing and appropriately adjusting the composite formulation, as well as selecting the manufacturing process, are crucial to achieve the best balance among stiffness, strength, and ductility in engineering components.

## 4. Discussion

The addition of diatomaceous earth, also referred to as biosilica, to the biopolymer polylactide (PLA), as well as the PLA itself, is a topic frequently addressed in experimental studies. In order to present a broader context and perspective on the issues discussed in this paper, it is necessary to compare them with those previously described in the literature. For clarity, Table 1 has been prepared, which shows the ranges and changes in basic parameters.

The level of crystallinity of a polymer has a significant influence on its mechanical properties. Polylactide is not a polymer that crystallizes easily, and only its stereocomplexes can achieve crystallinity levels of up to 55% [5,7]. To increase the level of crystallinity, annealing, stereochemical modifications in the polymer chain, or the use of nucleating additives that initiate crystallization can be applied [24]. Amorphous PLA is characterized by an inferior mechanical strength compared to its semi-crystalline variant. According to the literature, its tensile strength reaches a maximum of 30–60 MPa, with a stiffness of 0.4–3.5 GPa and elongation at break of 2–7% [1,2,25]. The semi-crystalline variant demonstrates better properties due to the presence of crystals that bind the polymer chains. The tensile strength increases to 50–70 MPa, and Young’s modulus rises to 2.7–4.2 GPa [1,2,4,21,25]. The elongation at break, due to the increased brittleness caused by the crystalline phase, falls within the range of 2–6% [2,4,21,25,26].

**Table 1:** Summary of mechanical properties of the PLA and PLA + DE composites and their changes depending on the processing method and silica filler content [2,4,18,21,22,23,26,27,28].

Index	Amorphous PLA	Semi-Crystalline PLA	Injection Molding (IM)	3D Printing (3DP)	Composite PLA + DE
**Young’s modulus** [GPa]	0.4–3.5	2.7–4.2	1.4–4.0	change from −25% to +10%	↗ (up to 15% DE) ↘ (over 15% DE)
**Tensile strength** [MPa]	30–60	55–70	45–70	change from −45% to +5%	↗ (up to 2.5% DE) ↘ (over 2.5% DE)
**Elongation at break** [%]	2–7	2–6	2–6	change from −35% to −25%	↗ (up to 2.5% DE) ↘ (over 2.5% DE)

The processing method has a strong influence on mechanical properties, especially on tensile strength. It significantly affects the shape of the stress–strain curve. Injection molding (IM) is considered the most favorable method in terms of the mechanical parameters of the product. This is related to the stability of the processing parameters, which is in line with a predefined temperature plan for the mold zones. Additionally, the pressure applied to the softened polymer should ensure a high packing density of the polymer mixture in the mold, resulting in the formation of tight polymer-chain bonds. Fused filament fabrication (FFF), in contrast, leads to the formation of voids between material paths and layers. Temperature variations and cooling of already formed layers reduce interlayer adhesion. Moreover, the FFF-printed parts are characterized by a high degree of anisotropy. The literature shows a wide range of variation in mechanical parameters. It is clear, however, that additive manufacturing reduces the elongation at break by 25–35% [23]. Most studies report a slight increase in Young’s modulus of printed PLA samples (up to around 10%), which is attributed to a more ordered polymer structure and increased crystallinity [2,26,28]. However, other data indicate a possible decrease of more than 25% [23]. Similar trends are reported for the tensile strength, with changes in the FFF ranging from a slight improvement (increase of 5%) [28] to significant deterioration (decrease of up to 45%) [23] compared to injection molding.

The form of the filler used is one of the main factors determining the final properties of the composite material. Diatomaceous earth can have different chemical and physical properties depending on its origin. The Perma-Guard diatomaceous earth used in this study is characterized by the presence of diatom shells with a maximum size of 50 µm. The material is mainly dominated by fractions ranging from 15 to 35 µm, with an average particle size of about 22 µm. The filler used does not differ in form from the diatomaceous earths used in other studies [22]. Additionally, based on microtomography studies [29], it can be stated that thermal processing of the PLA composite with diatomaceous earth (injection molding, extrusion, etc.) allows obtaining materials with good dispersion of the DE particles, with no agglomerates, which worsens the mechanical properties of the composite.

It should not be overlooked that the processing parameters, regardless of the method used, also influence the properties of the product. Higher processing temperatures or prolonged cooling times allow more time for the polymer to crystallize. Rapid cooling prevents the initiation of crystallization or results in the formation of small crystalline zones, while slower cooling or annealing leads to the development of larger crystals [2,4,23,25,27]. The literature suggests that additive manufacturing promotes higher levels of crystallization. This is likely due to the duration of the process. Injection molding, designed for mass production, is a fast polymer processing method that is focused on reducing the cycle time. Additive manufacturing, being a single-series production method, exposes the polymer to heat for a longer time, through a heated platform, a heated build chamber, or gradual cooling [23,27,28].

Polylactic acid (PLA) has gained significance as a versatile, biodegradable polymer that is suitable for both consumer and engineering applications. Its biological origin, processability, and compatibility with various fillers and reinforcements make it a sustainable alternative to conventional thermoplastics. Numerous examples in the literature describe the use of PLA as a matrix material, including composites with diatomaceous earth. Research has shown that introducing diatomaceous earth into PLA generally leads to a deterioration of basic mechanical properties, with the exception of the elastic modulus. The obtained results are consistent with previous studies. The tensile strength consistently decreases with an increasing content of diatomaceous earth in the material, at an estimated loss rate of 1–1.5 MPa per 1% by weight of increase in filler content. A similar trend is observed for elongation at break, which decreases by approximately 0.05–0.3% [21,22]. The only parameter that improves is Young’s modulus. Material stiffness increases due to the interaction of the filler by 0.02–0.08 GPa per 1% by weight of diatomaceous earth, with a noticeable slowdown in this trend at around 15% filler content [4,21,22].

However, the studies by Przekop et al. (2020) indicate that smaller amounts of diatomaceous earth (1–2%), lower than those tested in this paper, positively affect the tensile strength and elongation at break of the composite, increasing these properties by approximately 35% and 30%, respectively [30]. It should be noted, however, that the studies were conducted on conditioned (annealed) samples, which may have contributed to a significantly increased crystallinity of the composite material, without a noticeable negative impact from the poor PLA–DE adhesion, due to the low content of diatomaceous earth in the material [30].

The compatibility of diatomaceous earth with a polylactide matrix can be improved through appropriate pre-treatment. One example is the fractionation of the filler, which contributes to achieving better mechanical properties [22]. To ensure better adhesion while avoiding particle agglomeration, the surface of the filler can be modified using silane or lactic acid [4]. There are also known methods of pressure-based strengthening of polymer materials and polymer-based composites. Hydroextrusion allows for an increase in the material strength by approximately 40–50% [29].

It should be remembered that the final properties of the PLA + DE composite and other similar materials are always influenced by factors, such as the size and shape of filler particles, their prior preparation, as well as the processing method and time [2,4,28].

### 4.1. Analysis Summary

The results obtained from the research are consistent with the existing literature on the subject. Experimental studies confirm that the degree of crystallinity contributes to an increase in the tensile strength and Young’s modulus of polylactide. Crystallinity can be enhanced by annealing the material or by adding crystallization nucleators, such as diatomaceous earth.

Injection molding (IM) enables the production of more uniform elements, whereas additive manufacturing using FFF introduces numerous discontinuities, anisotropy, and porosity into the product, which result in a decrease in strength and ductility, although the stiffness of the product increases.

Young’s modulus increases with the addition and rising content of diatomaceous earth in the PLA matrix. Strength and ductility, however, decrease. The reason for these changes is the acceleration of the crystallization process and the formation of stress concentration points within the material. Problems related to adhesion or particle agglomeration can be reduced by applying surface modifications to the filler.

### 4.2. Trends in Polylactide Matrix Composites

Polylactic acid (PLA), due to its biodegradability, ease of processing (as a thermoplastic), and origin from renewable resources, has become a versatile material used across many industrial sectors. A major advantage is PLA’s adaptability in composite formulations. Its applications range from consumer goods to biomedical devices, offering solutions that align with sustainable development goals while meeting functional performance requirements.

In the packaging sector, pure PLA—both semi-crystalline and amorphous—is increasingly used as a feedstock for manufacturing biodegradable food containers and cutlery. Its compostability and food-contact safety, combined with the ability to form transparent, rigid objects, make it ideal for products such as coffee cups, salad boxes, or water-soluble trays. Beyond traditional processing methods, PLA has become one of the primary materials for 3D printing. Polymer modification is often achieved using biofillers. The aesthetic quality of the material and compliance with food-safety regulations are key advantages in this area of application [31].

In the biomedical field, PLA composites enriched with bioactive additives—such as hydroxyapatite, carbon fibers, or bioactive glass—are employed. These formulations enhance the osteoconductivity of the material while retaining its inherent biocompatibility and bioresorbability. Typical implementations include resorbable orthopedic implants, tissue-engineering scaffolds, and biodegradable drug-delivery systems. The temporary nature of these devices complements PLA’s degradation profile, providing therapeutic support during healing before naturally dissolving in the body [32].

Automotive engineering utilizes PLA that is reinforced with natural or carbon fibers as a sustainable alternative for producing interior vehicle components, such as door trims, glovebox liners, and insulating brackets. These composites, unlike pure PLA, meet mechanical and thermal performance standards. Their use supports OEMs’ eco-friendly initiatives, and their light weight contributes to fuel savings and reduced exhaust emissions [33].

Another growing application for PLA-based composites is in consumer electronics. Carbon-fiber or graphite reinforcements yield stiff, dimensionally stable structures with improved thermal and electromagnetic properties. These materials are used for laptop enclosures, phone cases, and drone components—often produced by additive manufacturing—where both mechanical performance and surface finish are critical [34].

PLA + carbon-fiber composites have also found early and broad use in the sports industry. They are successfully used to manufacture lightweight, semistructural parts, such as bicycle frame inserts, drone components, and bottle holder mounts. Thanks to PLA’s excellent compatibility with FFF printing, these composites enable geometrically customized production, facilitating both prototyping and short-run manufacturing of sports equipment tailored to individual users [35].

Leveraging PLA’s ease of processing, low warpage, and visual appeal, PLA-based materials are also used to develop educational models and teaching aids. The addition of wood flour, diatomaceous earth, chalk, or even copper makes PLA an excellent material for printing architectural models, gear assemblies, and educational demonstration kits. Such materials are inexpensive, safe for classroom use, and combine hands-on learning with an eco-friendly approach [36,37].

PLA and natural-fiber composites are further utilized in art and design. They yield products such as lampshades, 3D-printed furniture, or planters. Due to the eco-friendly nature of the feedstock, these items have minimal environmental impact while maintaining high aesthetic value. They are particularly well suited for small runs or bespoke interior design projects, imparting an artistic and unique character to each piece [37,38].

As can be observed, PLA composites that are reinforced with carbon fibers are of particular interest in engineering and industrial applications. Carbon fibers (CF) significantly and positively influence the mechanical properties of the material. Chopped fibers are typically used as reinforcement in materials that can subsequently be processed by all conventional methods [37,39]. Continuous fiber, on the other hand, can be employed in 3D printing by using specially modified FFF technology. During the additive manufacturing process, the carbon fiber is fed through a dedicated nozzle, introduced into the molten thermoplastic, and consolidated with the matrix, resulting in components with properties comparable to those of engineering-grade materials. PLA–CF material is characterized by a substantial increase in tensile strength and Young’s modulus. This reinforcing effect is particularly pronounced when continuous fiber is used [37,39]. A noticeable drawback of carbon-fiber composites is the reduction in elongation at break—by as much as 3% compared to the pure PLA matrix—making the material more brittle. PLA-based composites also feature hybrid reinforcement systems by combining carbon fibers with graphite flakes. This configuration not only enhances the mechanical properties but also positively affects tribological performance [40]. For engineering applications, such as the production of additively manufactured rotors, a PLA composite with graphene is used [41] with appropriate optimization of the extrusion process [41,42].

PLA-based composites, therefore, span a wide spectrum of applications, from artistic installations and medical bioimplants to purely engineering structures. This versatility underscores PLA’s potential: by selecting appropriate additives, performing chemical modifications, and applying suitable conditioning, one can tailor its properties to meet diverse engineering and functional requirements. However, the filler in the form of diatomaceous earth does not change the environmentally friendly nature of PLA due to its natural origin.

## 5. Conclusions

Polylactic acid (PLA), more broadly known as a polylactide biopolymer, is a widely used thermoplastic that has found applications across diverse industrial and scientific fields. An undeniable advantage is its ability to combine with a wide range of fillers and reinforcements in composite materials. This versatility allows for precise tailoring of the composite’s physical and mechanical properties to suit specific applications.

The conducted studies largely confirm the literature reports on polylactide–diatomaceous earth (PLA-DE) composites. Crystallinity is one of the key factors that determines a material’s mechanical and processing properties. The semi-crystalline variant, PLA Ingeo 4043D, exhibits the highest tensile strength, approaching the upper bound of 70 MPa, and Young’s modulus, ranging from 3.75 to 4.65 GPa, depending on the DE content and fabrication route. Amorphous variant PLE 0005-A, lacking the polymer-chain-bridging crystals, shows lower mechanical properties. However, biopolymer PLE 005-A is intended for applications requiring an amorphous variant.

Diatomaceous earth—a natural biosilica filler—increases composite stiffness at the expense of strength. This underscores the role of mineral fillers in stiffening the matrix. The literature’s report of slight strength improvements at low DE concentrations is attributed to diatomaceous particles acting as crystallization nucleators. However, at higher loadings, they become stress concentrators at the matrix–filler interface, exacerbated by particle agglomeration.

The manufacturing method is another critical factor that influences the final mechanical performance. Additive manufacturing—particularly FFF technology—makes extensive use of PLA and its composites. However, compared with injection molding, it significantly degrades ductility, rendering parts brittle. The literature often reports a decline in tensile strength as well, though this was not observed in our results, possibly due to a high extrusion ratio (115%), which improved inter-bead and interlayer fusion. Furthermore, 3D printing’s longer thermal cycle can increase crystallinity, but due to anisotropy and porosity, it still produces lower strength and ductility than the homogeneous injection-moulded parts.

An optimal balance of mechanical properties was achieved in the PLA + DE composites containing 5–10% by weight of diatomite, which was processed by injection molding. These composite samples showed increased stiffness while maintaining acceptable strength and ductility. It has been recently shown that to further enhance the performance of polylactide and its based materials, processes based on hydroextrusion can be applied.

## Data Availability

The data supporting this study’s findings are available from the corresponding author upon request.

## Abbreviations

The following abbreviations are used in this manuscript:

PLA	Polylactide
DE	Diatomaceous earth
IM	Injection molding
3DP	3D printing/additive manufacturing
FFF	Fused Filament Fabrication
E	Young’s modulus
MFR	Melt Flow Rate
ROP	Ring-opening polymerization
PLLA	Poly-L-lactic acid
PDLA	Poly-D-lactic acid
PDLLA	Poly-D,L-lactic acid
scPLA	Stereocomplex polylactide
OEM	Original Equipment Manufacturer
CF	Carbon fibers
